# Comprehensive assessment of knee joint synovitis at 7 T MRI using contrast-enhanced and non-enhanced sequences

**DOI:** 10.1186/s12891-020-3122-y

**Published:** 2020-02-21

**Authors:** Christoph Treutlein, Tobias Bäuerle, Armin M. Nagel, Ali Guermazi, Arnd Kleyer, David Simon, Georg Schett, Tobias Hepp, Michael Uder, Frank W. Roemer

**Affiliations:** 1 Department of Radiology, Friedrich-Alexander University Erlangen-Nuremberg (FAU) and Universitätsklinikum Erlangen, Maximiliansplatz 3, 91054 Erlangen, Germany; 2 Institute of Medical Physics, University of Erlangen, Friedrich-Alexander-Universität Erlangen-Nürnberg (FAU), Erlangen, Germany; 3 Division of Medical Physics in Radiology, German Cancer Research Centre (DKFZ), Heidelberg, Germany; 4 Quantitative Imaging Center (QIC), Department of Radiology, Boston University School of Medicine, 820 Harrison Avenue, FGH Building, 3rd Floor, Boston, MA 02118 USA; 5 Department of Radiology, Veterans Affairs Boston Healthcare System, 1400 VFW Parkway, Suite 1B105, Boston, MA 02132 USA; 6 Department of Medicine 3, Rheumatology and Immunology, Friedrich-Alexander University of Erlangen-Nuremberg (FAU) and Universitätsklinikum Erlangen, Ulmenweg 18, 91054 Erlangen, Germany; 7 Department of Medical Biometry, Informatics and Epidemiology, Faculty of Medicine, University of Bonn, Sigmund-Freud-Straße 25, 53105 Bonn, Germany; 8 Institute of Medical Informatics, Biometry and Epidemiology, Friedrich-Alexander University of Erlangen-Nuremberg, Waldstraße 6, 91054 Erlangen, Germany

**Keywords:** MRI, Knee, Synovitis, 7 T, Ultra-high field MRI

## Abstract

**Background:**

Seven T ultra-high field MRI systems have recently been approved for clinical use by the U.S. and European regulatory agencies. These systems are now being used clinically and will likely be more widely available in the near future. One of the applications of 7 T systems is musculoskeletal disease and particularly peripheral arthritis imaging. Since the introduction of potent anti-rheumatic therapies over the last two decades MRI has gained increasing importance particularly for assessment of disease activity in early stages of several rheumatic disorders. Commonly gadolinium-based contrast agents are used for assessment of synovitis. Due to potential side-effects of gadolinium non-enhanced techniques are desirable that enable visualization of inflammatory disease manifestations. The feasibility of 7 T MRI for evaluation of peripheral arthritis has not been shown up to now. Aim of our study was to evaluate the feasibility of contrast-enhanced (CE) and non-enhanced MRI at 7 T for the assessment of knee joint synovitis.

**Method:**

Seven T MRI was acquired for 10 patients with an established diagnosis of psoriatic or rheumatoid arthritis. The study pulse sequence protocol was comprised of a sagittal intermediate-weighted fat-suppressed (FS), axial fluid-attenuated inversion recovery (FLAIR) FS, sagittal 3D T1-weighted dynamic contrast enhanced (DCE) and an axial static 2D T1-weighted FS contrast-enhanced sequence (T1-FS CE). Ordinal scoring on non-enhanced (Hoffa- and effusion-synovitis) and enhanced MRI (11-point synovitis score), and comparison of FLAIR-FS with static T1-FS CE MRI using semiquantitative (SQ) grading and volume assessment was performed. For inter- and intra-reader reliability assessment weighted kappa statistics for ordinal scores and intraclass correlation coefficients (ICC) for continuous variables were used.

**Results:**

The total length of study protocol was 15 min 38 s. Different amounts of synovitis were observed in all patients (mild: *n* = 3; moderate: *n* = 5; severe: *n* = 2). Consistently, SQ assessment yielded significantly lower peripatellar summed synovitis scores for the FLAIR-FS sequence compared to the CE T1-FS sequence (*p* < 0.01). FLAIR-FS showed significantly lower peripatellar synovial volumes (*p* < 0.01) compared to CE T1-FS imaging with an average percentage difference of 18.6 ± 9.5%. Inter- and intra-reader reliability for ordinal SQ scoring ranged from 0.21 (inter-reader Hoffa-synovitis) to 1.00 (inter-reader effusion-synovitis). Inter- and intra-observer reliability of SQ 3D-DCE parameters ranged from 0.86 to 0.99.

**Conclusions:**

Seven T FLAIR-FS ultra-high field MRI is a potential non-enhanced imaging method able to visualize synovial inflammation with high conspicuity and holds promise for further application in research endeavors and clinical routine by trained readers.

## Background

Seven T MRI has evolved rapidly over the past decade [1]. With clearance for clinical use by the U.S. and European regulatory agencies recently, 7 T systems are already used clinically and will potentially become more widely available [2].

MRI has made an important clinical impact in the assessment of inflammatory arthritis particularly since the introduction of effective anti-rheumatic therapies [3–5]. MRI is particularly suited regarding visualization of hallmark features of inflammation like synovitis and osteitis characterizing early disease and being predictive of progression [6, 7]. Recommended imaging protocols on standard 1.5 T and 3 T systems include T1- and T2-weighted fat suppressed spin echo sequences and the use of contrast-enhanced (CE) MRI for the evaluation of synovitis [8–10]. Experience regarding feasibility of CE imaging at 7 T to date is limited and no data is available for CE imaging of synovitis at 7 T [11, 12]. Due to its higher signal-to-noise ratio 7 T MRI has potential advantages particularly in the assessment of early disease and likely will be used more frequently in a clinical context in the near future [1].

In addition, given potential side effects of gadolinium and reports on gadolinium deposition in the brain, novel non-enhanced techniques replacing CE imaging are desirable that enable differentiation of intraarticular joint fluid from synovial thickening as two distinct features of joint inflammation [13]. Fluid attenuated fat suppressed- (FLAIR–FS) and double inversion-recovery (DIR) sequences seem promising in this regard and preliminary feasibility of both has been shown for 3 T systems [14, 15].

Thus, the aim of this study was to assess clinical feasibility of comprehensive synovitis assessment of the knee joint applying non-enhanced and CE sequences on a 7 T MRI system in patients with established inflammatory arthritis.

## Methods

### Patients

This prospective study included 10 consecutive patients with an established diagnosis of either psoriatic (according to Classification Criteria for Psoriatic Arthritis - CASPAR) or rheumatoid arthritis (according to American College of Rheumatology - ACR/European League against Rheumatism -EULAR 2010 criteria). Patients with an acute episode of a swollen and painful knee joint were recruited from the rheumatologic outpatient clinic of Universitätsklinikum Erlangen between February and August 2018. Written informed consent was obtained for this ethics board-approved investigation (Local IRB number: AZ_189_15B). Exclusion criteria were any metallic, electronic or magnetic implants, any tattoos, renal insufficiency (defined as an estimated glomerular filtration rate < 60 ml/min/1.73 m^2^) and contraindications for contrast administration [16]. In addition, women who were pregnant or planned to be pregnant were not included. Potential temporary bioeffects of the 7 T system including nystagmus, nausea, and vertigo were included in the consent form.

### Image acquisition

All MRI examinations were performed on a 7 T platform (Magnetom Terra, Siemens Healthineers, Erlangen, Germany) with a dedicated 1-channel transmit and 28-channel receive knee coil (Quality Electrodynamics, Mayfield Village, OH). The MRI protocol comprised a sagittal 2D intermediate-weighted fat suppressed (IW-FS) turbo spin echo, an axial 2D FLAIR-FS sequence, a time-resolved sagittal 3D T1-weighted fast low angle shot (FLASH) sequence for dynamic contrast-enhanced (DCE) imaging acquired over 200 s and an axial 2D T1-weighted FS CE (T1-FS CE) sequence. Detailed sequence parameters are provided in Table 1.

**Table 1:** Sequence protocol for synovitis assessment at 7 T

Parameter	IW-FS	FLAIR-FS	3D-DCE	T1-FS CE
Type of sequence	TSE	TSE	FLASH	TSE
Voxel size (mm)	0.37 × 0.37 × 2.5	0.36 × 0.36 × 2.5	1.1 × 1.1 × 1.1	0.36 × 0.36 × 2.5
Orientation	sagittal	axial	sagittal	axial
FOV (mm)	160	160	160	160
Matrix	432	448	144	448
Bandwith (Hz/Pixel)	227	286	285	219
Number of slices	31	44	104	33
Slice thickness (mm)	2.5	2.5	1.1	2.5
Number of acquisitions (NEX)	1	1	22 (every 9.8 s)	1
TR (ms)	4480	9000	4.5	1580
TE (ms)	36	86	1.77	12
TI (ms)	n/a	2000	n/a	n/a
Flip angle (°)	180	180	15	180
Fat suppression	Frequency selective fat saturation	Frequency selective fat saturation	Water excitation	Frequency selective fat saturation
PAT mode / acc. Factor	GRAPPA / 2	GRAPPA / 3	GRAPPA / 3	GRAPPA / 2
Dimension	2D	2D	3D	2D
Echo trains per slice	76	7	n/a	118
Scan time (min:s)	3:15	4:32	3:40	3:11
Overall scan time (min:s)	15:38			

Abbreviations: *IW-FS* Intermediate weighted fat-suppressed sequence, *T1-FS CE T1-weighted fat suppressed contrast-enhanced sequence*, *FLAIR-FS* Fluid attenuated inversion recovery fat suppressed sequence, *3D-DCE* Three dimensional dynamic contrast enhanced sequence, *TSE* Turbo spin echo, *FLASH* Fast low-angle shot sequence, *FOV* Field of view, *TR* Repetition time, *TE* Echo time, *TI* Inversion time, *PAT* Parallel imaging technique, *GRAPPA* GeneRalized Autocalibrating Partial Parallel Acquisition

The development of the FLAIR-FS sequence was focused at nulling the signal from intraarticular fluid and was tested in a preliminary series with a patient who was not part of the final study sample (Appendix 1).

The DCE sequence was acquired with 22 repetitive measurements every 9.8 s after i.v. administration of 0.1 mmol/kg Gadobutrol (Gadovist® 1.0 mmol/ml, Bayer Vital, Leverkusen, Germany). The contrast agent was injected manually starting just prior to the beginning of the first measurement at a rate of approximately 1 ml/s.

### Image analysis

#### Semiquantitative evaluation

**Fig. 1:**
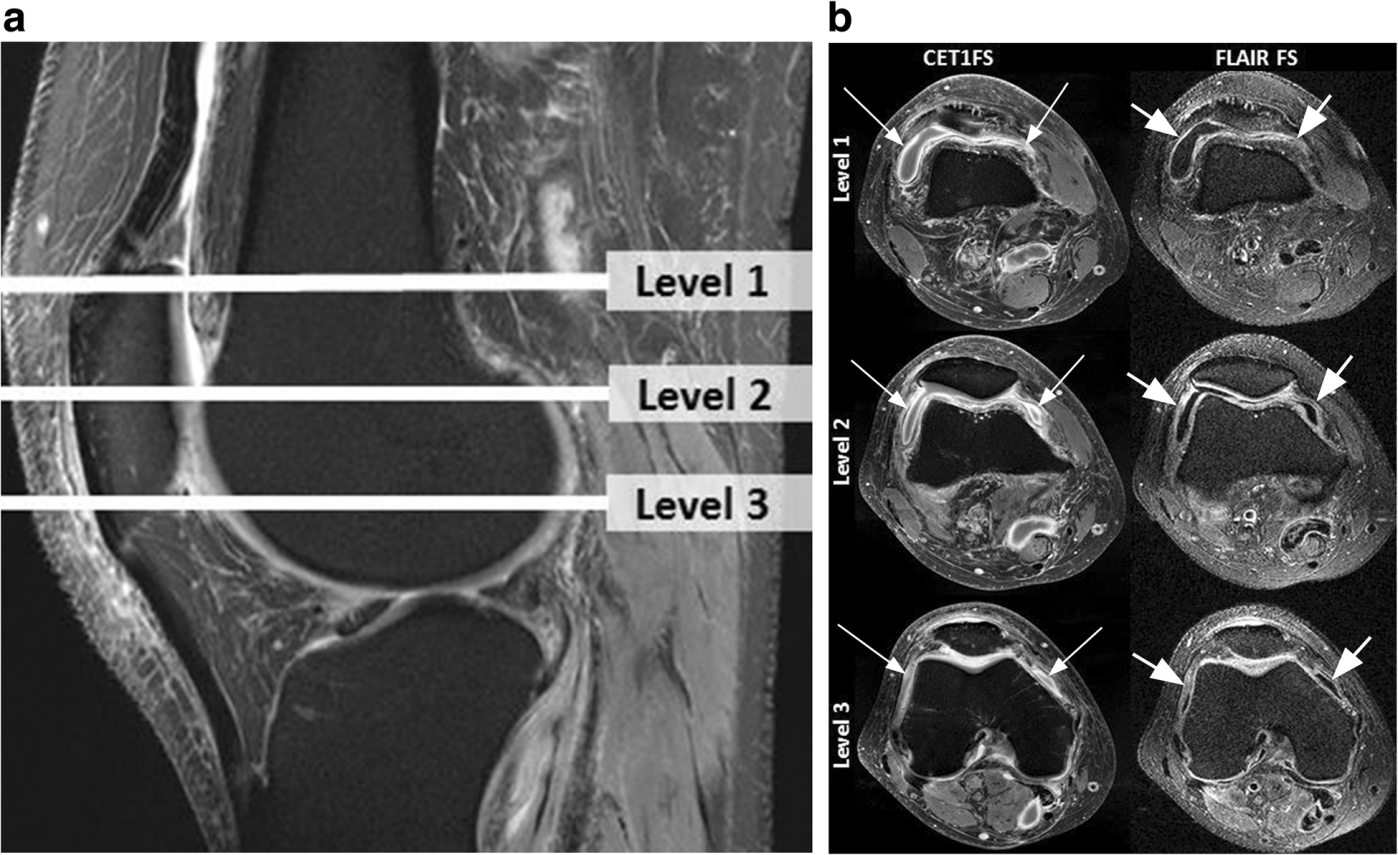
Anatomic coverage and visualization of synovitis on contrast-enhanced and non-enhanced sequences. **a** Sagittal intermediate-weighted fat suppressed (IW-FS) image shows the different axial levels that are depicted in **b** Level 1 represents the transverse slice at the level of the superior patellar pole. Level 2 is defined by the mid-point of the patella in the cranio-caudal direction and Level 3 is representing the transverse slice at the level of the inferior patellar pole. **b** Corresponding transverse image pairs of contrast-enhanced T1-weighted fat suppressed (T1-FS CE) and non-enhanced fluid attenuated inversion recovery fat suppressed (FLAIR-FS) sequences at each of the three levels of the femoro-patellar joint. Left figure column depicts the T1-weighted enhanced images with synovial thickening and contrast-enhancement at all levels (long arrows). Figure parts in the right figure column show corresponding FLAIR-FS images with synovitis being depicted in similar fashion as hyperintense with corresponding thickening of the synovial tissue at all levels (short arrows). Level 2 was used for semiquantitative assessment of peripatellar synovitis according to reference 16. Note that FLAIR images show synovial thickening to a somewhat lesser extent compared to T1-FS CE images

MRI readings were performed by a radiologist with 14 years’ (*F.W.R.*) experience in semiquantitative MRI assessment of knee disorders blinded to clinical diagnosis. First, signal alterations in the intercondylar region of Hoffa’s fat pad were scored on non-enhanced IW-FS images on a scale of 0–3 as a surrogate for synovial thickening termed ‘Hoffa-synovitis’. Joint effusion (‘effusion-synovitis’) was graded on a scale of 0–3 in terms of the estimated maximal distention of the synovial cavity using the same sequence [17]. Whole knee synovitis was scored on the T1-FS CE sequences using a modification of a validated scoring system at 11 sites of the joint from 0 to 3 [18]. In addition to the original description of this instrument a grade 3 was introduced representing synovitis of > 5 mm thickness with grade 2 representing synovitis of > 4 and ≤ 5 mm. Reason for this adaptation was that the current patient sample had a high probability of severe synovitis. For definition of severity of whole-knee synovitis the scores of the 11 sites were summed and categorized as follows: 0–5 normal or equivocal; 6–9 mild; 10–13 moderate and ≥ 14 severe synovitis. In addition, synovitis was assessed at the medial and lateral peripatellar recesses on axial FLAIR-FS images in identical fashion (Fig. 1). MRIs were evaluated using eFilm software (Version 4.2.0, Merge Healthcare Inc., Chicago, IL). Reliability readings were performed by the same reader and a second radiologist with 20 years’ experience in standardized knee joint assessment (*A.G.*) after a 4-week interval.

#### Volume assessment

**Fig. 2:**
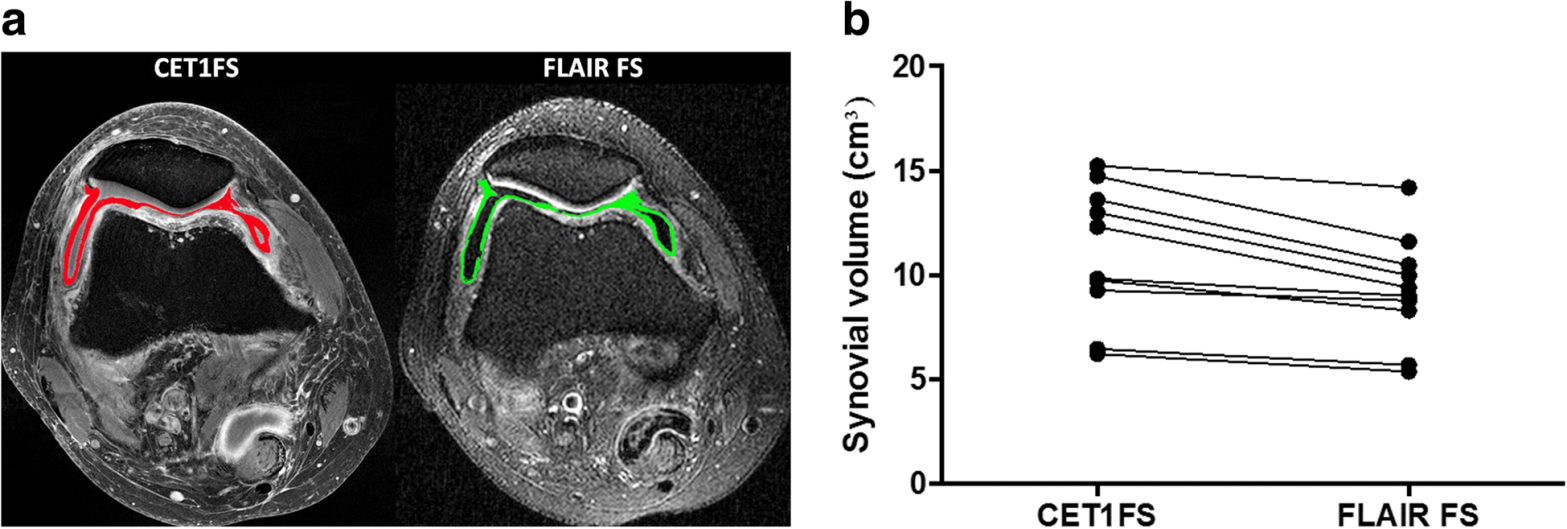
Volumetric synovitis assessment. **a** Left part of figure shows transverse T1-weighted fat suppressed contrast enhanced (T1-FS CE) image at Level 2 (see Fig. 1a). Segmented synovium is depicted in red. Note perisynovitic inflammatory infiltration of soft tissues. Right part of figure shows corresponding FLAIR-FS image with synovium being segmented at the same level and colored in green. Volume assessment was performed for all slices between Level 1 and Level 3 (inclusive of Level 1 and 3) in the cranio-caudal dimension. **b** Comparison of volume measurements for T1-FS CE images and FLAIR-FS for all knees analyzed. Note the persistently lower volume assessments for FLAIR-FS. Peripatellar synovial volume obtained from axial FLAIR-FS images was statistically significantly lower (*p* < 0.01) compared to T1-FS CE images ranging between 5.37 and 11.59 cm^3^ compared to 6.22 and 14.74 cm^3^ for T1-FS CE images. The mean difference between the two sequences was 19% less synovial volume for FLAIR-FS

Synovial volume was determined based on manual segmentation of all axial slices between the superior and inferior patellar pole on axial T1-FS CE and FLAIR-FS images using Aycan-OsiriX (v.2) software (aycan Digitalsysteme GmbH, Würzburg, Germany) with Chimaera segmentation plugin (Chimaera GmbH; Erlangen, Germany). All segmentations were performed by a trained radiologist (C.T.) with 4 years’ experience in MSK MRI (Fig. 2a). Reliability segmentations were performed by the initial reader and a second radiologist with 12 years’ experience in MSK MRI *(T.B.)* after an interval of 4 weeks in identical fashion.

#### DCE measurements

**Fig. 3:**
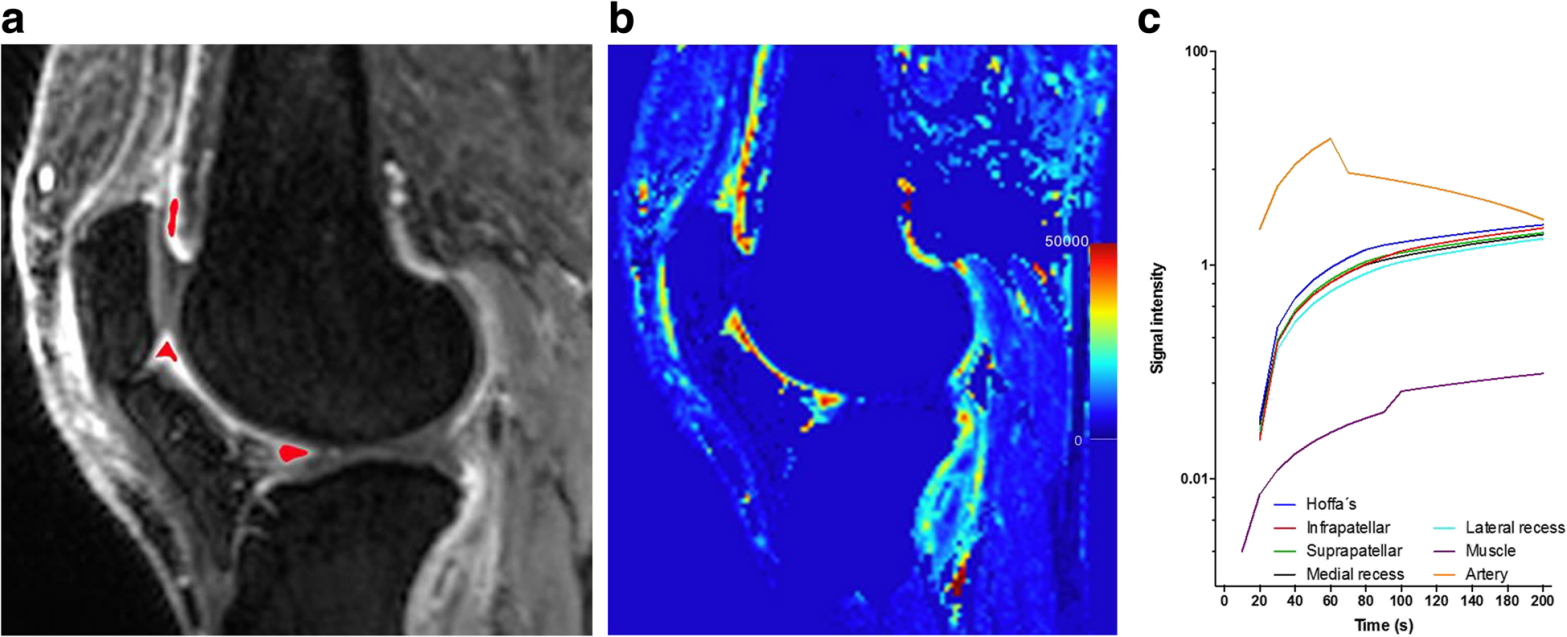
Dynamic contrast enhanced MRI. **a** Example of a sagittal T1-weighted dynamic contrast enhanced (DCE) image with demonstration of ROI placement in the last acquired image series at the most posterior border of Hoffa’s fat pad, adjacent to the inferior patellar pole and at the prefemoral fat pad. Regions of interest (ROIs) were centered within the synovial tissue, joint effusion or other articular structures were not included. **b** iAUC based calculation of parametric maps over time (color-coding represents arbitrary units (a.u.) of the iAUC from dynamic contrast-enhanced MRI ranging from blue to red (0–50.000 a.u.). **c** Typical enhancement curves from a single patient for the different ROIs show steep enhancement between 20 and 40 s followed by continued but much slower enhancement after 40 s

Five regions of interest (ROIs) were drawn manually at an anatomical midline location in the infrapatellar, the intercondylar and prefemoral regions, in the popliteal artery and the gastrocnemius muscle (Fig. 3a). The drawing procedure was performed by the reader who created the manual segmentations (*C.T.*). For each ROI perfusion variables were extracted as follows: average slope in the initial 30 s of measurement (‘wash-in’), average slope in the last 30 s of measurement (‘wash-out’), time-to-peak enhancement (TTP), peak enhancement ratio (PE) defined as (S_max_ – S_0_)/S_0_ (S_max_ - *maximum signal intensity* and S_0_ - *pre-contrast signal intensity*), and initial area under the curve (iAUC) defined for the first 60 s (Fig. 3b). Time intensity curve shapes were created for the different ROIs and plotted against the reference curves of artery and muscle (Fig. 3c). Intra- and inter-reader reliability was performed after the same 4 week interval by the same readers who assessed the volumes.

### Statistical analysis

Assumptions of normality were checked by visual inspection of quantile-quantile-plots with log-transformation. The paired t-test to evaluate differences of synovial volumes on non-enhanced and CE sequences was applied. In addition, Pearson’s correlation coefficients were calculated to reflect the linear correlation for assessment using the two different sequences. Agreement was assessed using weighted kappa statistics for ordinal measures and intraclass correlation coefficients (ICC) for continuous measures. The interpretation of the reliability results was based on the suggestions by Landis and Koch for SQ parameters (w-kappa) and by Koo and Mae for continuous variables (ICC) [19, 20]. A *p* value < 0.05 was considered statistically significant. All analyses were performed using R version 3.5.2 (R Foundation for Statistical Computing, Vienna, Austria).

## Results

Ten patients with established psoriatic (*n* = 4) or rheumatoid arthritis (*n* = 6) were included. All patients had active arthritis. Mean disease duration was 3.2 years (range 6 months to 18 years). Two patients were women; mean age was 48.4 ± 13.7 years. Mean body mass index (BMI) was 25.8 ± 2.4 kg/m^2^, C-reactive protein serum levels at the time of imaging were 12.3 mg/l (range 0 to 58.1 mg/l, normal value < 5 mg/l).

Our study protocol included four sequences that were acquired in a total scan time of just over 15 min. The sagittal IW-FS sequence was acquired in slightly over 3 min and adding axial and coronal IW-FS sequences - as is commonly the case in the clinical routine - would result in a total imaging time for a standard clinical protocol of 17 to 18 min, including static CE and DCE MRI while omitting the experimental FLAIR-FS sequence.

The summed synovitis scores from 11-point scoring in T1-FS CE images classified 3 patients as having mild, 5 as moderate and 2 as having severe whole joint synovitis. The peripatellar synovitis score obtained from T1-FS CE and FLAIR-FS images at two sites ranged from 0 to 6 (Table 2**)**. SQ assessment yielded significantly lower peripatellar summed synovitis scores for the FLAIR-FS sequence (mean 2.4 ± 1.6) compared to the CE T1-FS sequence (mean 3.3 ± 1.6, *p* < 0.01). However, the results were highly correlated with Pearson’s r of 0.938 for SQ evaluation and 0.948 for volume assessment.

**Table 2:** Semiquantitative, volumetric and dynamic contrast-enhanced MRI assessment at 7 T MRI

Parameter	Patient										Intra-reader Agreement ^d^	Inter-reader Agreement ^d^
	1	2	3	4	5	6	7	8	9	10		
Semiquantitative scoring												
Hoffa-Synovitis-score (IW-FS) ^a^	1	1	1	1	2	3	3	1	0	1	0.76 [0.45 to 1.05]	0.21 [0.18 to 0.61]
Effusion-Synovitis-score (IW-FS) ^a^	3	2	2	3	3	2	2	3	2	2	0.64 [0.20 to 1.08]	1.00 [1.00 to 1.00]
11-site score (summed; T1-FS CE) ^b^	11	12	18	8	18	6	12	6	9	13	0.93 [0.77 to 0.98]	0.93 [0.75 to 0.98]
Synovitis severity ^b^	moderate	moderate	severe	mild	severe	mild	moderate	mild	moderate	moderate	n/a	n/a
Peripatellar T1-FS CE(2 sites) ^c^	3	4	6	3	6	2	3	1	2	3	0.89 [0.74 to 1.02]	0.87 [0.75 to 1.02]
Peripatellar FLAIR-FS(2 sites) ^c^	2	4	5	2	4	1	2	0	1	3	0.77 [0.59 to 0.94]	0.68 [0.49 to 0.88]
Volume (cm^c^)												
T1-FS CE	14.74	13.61	15.24	12.98	9.27	12.30	6.22	6.46	9.75	9.83	0.93 [0.69 to 0.98]	0.98 [0.93 to 0.99]
FLAIR-FS	11.59	10.47	14.18	10.01	8.78	9.40	5.37	5.70	8.31	9.01	0.91 [0.60 to 0.98]	0.88 [0.59 to 0.97]
3D-DCE												
Wash-in	1.12 ± 0.81	0.67 ± 0.41	2.23 ± 1.79	1.06 ± 1.02	1.84 ± 2.66	2.51 ± 2.98	−0.25 ± 1.59	1.38 ± 1.55	1.72 ± 1.21	2.41 ± 2.82	0.97 [0.99 to 0.99]	0.87 [0.78 to 0.92]
Wash-out	0.17 ± 0.24	00.42 ± 0.36	0.11 ± 0.37	0.19 ± 0.38	− 0.02 ± 0.50	0.15 ± 0.71	0.68 ± 0.39	0.13 ± 0.36	0.11 ± 0.36	− 0.01 ± 0.59	0.95 [0.89 to 0.97]	0.91 [0.84 to 0.94]
TTP	1.04 ± 0.44	0.93 ± 0.68	0.86 ± 0.58	0.96 ± 0.30	0.93 ± 0.38	1.03 ± 0.29	1.29 ± 0.51	0.86 ± 0.34	0.70 ± 0.22	0.63 ± 0.12	0.82 [0.71 to 0.89]	0.79 [0.67 to 0.88]
PE	0.93 ± 1.49	1.68 ± 1.12	2.19 ± 1.35	2.56 ± 2.94	1.78 ± 1.89	5.32 ± 6.86	3.36 ± 3.68	2.15 ± 2.33	1.93 ± 1.03	2.55 ± 2.49	0.99 [0.98 to 0.99]	0.86 [0.78 to 0.92]
iAUC	0.67 ± 0.53	0.69 ± 0.55	1.16 ± 0.81	1.09 ± 1.55	0.82 ± 1.07	1.91 ± 2.58	0.88 ± 1.02	0.85 ± 1.10	1.00 ± 0.65	1.69 ± 2.20	0.99 [0.99 to 0.99]	0.88 [0.80 to 0.93]

^a^ Hoffa- and effusion synovitis was scored semiquantitatively from 0 to 3 on the sagittal IW-FS sequence

^b^ Summed 11-site whole-joint semiquantitative score (from 0 to 3): the medial and lateral peripatellar recess, suprapatellar, infrapatellar, intercondylar, medial and lateral perimeniscal, and adjacent to the anterior and posterior cruciate ligaments. If knees presented with Baker’s cysts or loose bodies, these two sites were scored in addition. For assessment of whole-knee synovitis the scores of the 11 sites were summed and categorized: 0–5 normal or equivocal synovitis; 6–9 mild synovitis; 10–13 moderate synovitis and ≥ 14 severe synovitis adapted from reference 16

^c^ Scored at the medial and lateral peripatellar recess from 0 to 3 at level 2 (see Fig. 1a) - adapted from reference 16

^d^ Weighted Kappa statistics were used for ordinal scores and intraclass correlation coefficients (ICC) were used for continuous variables

Data are means ± standard deviations, unless indicated otherwise. Numbers in parentheses are 95% confidence intervals

Abbreviations: *T1-FS CE* T1-weighted fat suppressed contrast-enhanced sequence, *FLAIR-FS* Fluid attenuated inversion recovery fat suppressed sequence, *3D-DCE* Three dimensional dynamic contrast enhanced sequence, *S*_*0*_ Pre-contrast signal intensity, *S*_*max*_ Maximum signal intensity, *a.u* Arbitrary unit, Wash in: average slope during initial (first 30 s) enhancement (a.u/min); Wash out: average slope in the washout (last 30 s) phase (a.u/min); *TTP* Time to peak (s), *PE* Peak enhancement ratio (S_max_ – S_0_)/S_0_; iAUC Initial area under the curve (first 60s, a.u.min)

Peripatellar synovial volume obtained from axial FLAIR-FS images was statistically significantly lower (*p* < 0.01) compared to T1-FS CE images ranging between 5.37 and 11.59 cm^3^ compared to 6.22 and 14.74 cm^3^ for T1-FS CE images (Table 2). The mean difference between the two sequences was 18.6% ± 9.5% less synovial volume for FLAIR-FS (Fig. 2b).

Time intensity curves showed steep initial enhancement in the first 30 s with continued but much slower enhancement at later time points as shown in Fig. 3c. Detailed DCE measurements for each patient are presented in Table 2. Mean wash in ratio was 1.46 ± 2.05, mean wash-out 0.20 ± 0.49, TTP 92 ± 45 s, peak enhancement ratio 2.55 ± 3.18, mean iAUC 1.08 ± 1.43.

Inter- and intraobserver agreement for SQ scoring ranged from 0.21 (inter-reader Hoffa-synovitis) to 1.00 (inter-reader effusion-synovitis). For SQ assessment three of the ten (30%) analyzed parameters were considered in the category of substantial agreement while five (50%) reflected almost perfect agreement. For volume assessment ICCs ranged from 0.88 (FLAIR FS inter-reader) to 0.98 (T1-FS CE inter-reader). Inter- and intraobserver agreement of SQ 3D-DCE parameters ranged from 0.79 to 0.99. Of the 14 analyzed quantitative parameters for volume and DCE assessment eight (57%) reflected excellent agreement while the remaining 6 (43%) were considered to reflect good agreement. Intra- and inter-reader agreement results are presented in detail in Table 2.

## Discussion

In our proof-of-concept study of 10 patients with established inflammatory arthritis and a swollen and painful knee joint we could show that comprehensive synovitis assessment is feasible at 7 T applying the reference standard for synovitis visualization, i.e. dynamic and static contrast-enhanced sequences. In addition, we were able to demonstrate in a confirmatory fashion that non-enhanced synovitis assessment using inversion recovery techniques seems promising also at ultra-high field MRI [14, 15]. Semiquantitative image assessment using a modified validated scoring instrument can be performed in a comparable fashion to standard systems. Volume quantification and DCE MRI showed good to excellent agreement between readers.

Our study protocol included four sequences that were acquired in a total scan time of just over 15 min. The sagittal IW-FS sequence was acquired in slightly over 3 min and adding axial and coronal IW-FS sequences - as is commonly the case in the clinical routine - would result in a total imaging time for a standard clinical protocol of 17 to 18 min, including static CE and DCE MRI while omitting the experimental FLAIR-FS sequence. We used a somewhat higher resolution (2.5 mm slice thickness, matrix ranging from 432 × 432 to 448 × 448) than is commonly the case for clinical 1.5 T and 3 T examinations (these commonly use 3 mm slice thickness with a matrix of 256 × 256) resulting in comparable scan time for the entire protocol. Potentially, the higher field strength at 7 T may be used to reduce total scan time at a similar resolution currently applied in clinical systems at 1.5 or 3 T [21].

Our aim was not to show superiority of 7 T MRI over established clinical systems but to demonstrate feasibility of comprehensive synovitis assessment at 7 T. With recent clearance for clinical application by regulatory authorities in the U.S. and Europe 7 T holds promise to be used in a broader clinical context. Beyond commonly applied assessment methodologies as applied in the current study 7 T has potential to provide additional information regarding synovitis evaluation not available at lower field strengths. These are likely to be based on metabolic approaches such as chemical exchange saturation transfer (CEST) methods or non-proton imaging that may add to our understanding of inflammatory joint disorders [1, 22, 23].

Using a slight modification of a validated semiquantitative scoring system we found good agreement for intra-reader (w-kappa 0.93, 95% confidence interval (CI) [0.77–0.98]) as well as inter-reader (w-kappa 0.93, 95% CI [0.75–0.98]) reliability. These values are comparable to the original description of the scoring system (performed at 1.5 T MRI) where authors reported agreement of 0.67–1.00 for reader 1 (weighted κ) and 0.60–1.00 for reader 2 for individual sites [18]. Inter-reader agreement was 0.67–0.92 (w-kappa, intrareader reliability). Intra-reader reliability (intraclass correlation coefficient) was 0.98 and 0.96 for each reader and inter-reader agreement (ICC) was 0.94 for summed synovitis scores across all 11 locations. Currently no data has been published using the 11-point synovitis score at 3 T MRI. Regarding DCE measures Axelsen et al. assessed responsiveness to treatment and reliability of DCE MRI in 10 rheumatoid arthritis knee joints on a 1.5 T system and reported high intra- and inter-reader reliabilities of the dynamic parameters with ICC values ranging between 0.96 to 1.00 [24]. Using a semi-automated method for synovial volume assessment Perry et al. described excellent intraobserver (ICC 0.99, 95% confidence interval (CI) 0.98–0.99) and good interobserver agreement 0.83 [95% CI 0.58–0.94]) on a 1.5 T system for 12 patients with knee osteoarthritis [25]. As for SQ assessment no data has been published specifically assessing reliability on 3 T systems.

We also applied non-enhanced assessment using an inversion recovery-based sequence, which has recently been described for 3 T MRI. Yoo et al. used the same validated SQ scoring system as we did in our study to evaluate peripatellar synovitis using CE MRI as the reference standard [15]. Our study expanded that work in that we also included volume assessment with FLAIR-FS. We found that these volumes consistently underestimated the true amount of synovitis when regarding CE MRI as the reference standard. An explanation may be that T1-FS CE overestimates true amount of synovitis owing to diffusion of contrast material into the joint cavity beyond the actual synovial lining [26]. On the other hand Son et al. reported a persistently greater synovial thickness measured on the DIR sequence compared to T1-FS CE suggesting that use of non-enhanced MRI may require a correction factor whenever estimation of true synovial volume is required [14].

Our study has limitations. This is a cross-sectional study on a small and highly selected sample and reported findings are not necessarily representative for larger populations. We used a standard dose of 0.1 mmol/kg Gadobutrol for CE imaging. At 7 T MRI the T1 relaxivities of Gadolinium-based contrast agents are lower than those at 3 T [27]. Nevertheless, preliminary work in brain tumors suggested that potentially half of the routine dose may be sufficient at 7 T, since also the T1 relaxation times of tissues change with field strength [11]. Future studies will have to show if lower doses may yield comparable results regarding synovitis visualization. Semiquantitative and heuristic DCE analyses were used instead of a pharmacokinetic DCE model since these methods are simple to implement, and robust in their performance, which would make implementation in a clinical setting feasible including monitoring of therapy response [28].

## Conclusions

In summary we could show that non-enhanced and CE synovitis assessment at 7 T MRI is clinically feasible and common semiquantitative and quantitative approaches to evaluate synovial characteristics can be obtained in reliable fashion at 7 T MRI. FLAIR-FS imaging is a promising non-enhanced imaging method able to visualize synovial inflammation and seems to support potential applicability in clinical studies and the routine by trained readers also at 7 T.

## Supplementary information

**Additional file 1.** Fluid attenuated inversion recovery fat suppressed imaging at 7T. The development of the FLAIR-FS sequence was focused at nulling the signal from intraarticular fluid in order to achieve an optimized image contrast between fluid and hyperintense synovium. In a preliminary series with a patient who was not part of the final study sample, a sequential experiment with inversion time values (TI) from 1800 to 2600 ms was performed and the most appropriate TI-value for fluid attenuation and differentiation between fluid and synovium was determined visually at 2000 ms.

## Data Availability

The datasets used and/or analyzed during the current study available from the corresponding author on reasonable request.

## Abbreviations

7 T: 7 Tesla
MRI: Magnetic resonance imaging
FS: Fat suppressed
CE: Contrast-enhanced
FLAIR: Fluid-attenuated inversion recovery
DCE: Dynamic contrast enhanced
T1-FS CE: T1-weighted FS contrast-enhanced sequence
SQ: Semiquantitative
ICC: Intraclass correlation coefficients
CASPAR: Classification criteria for psoriatic arthritis
ACR: American college of rheumatology
EULAR: European league against rheumatism
IRB: Institutional review board
MSK: Musculo-skeletal
ROI: Region of interest
TTP: Time-to-peak enhancement
PE: Peak enhancement ratio
iAUC: Initial area under the curve
