# Supplementary material for: Comprehensive assessment of knee joint synovitis at 7 T MRI using contrast-enhanced and non-enhanced sequences
Source: BMC Musculoskelet Disord. 2020 Feb 21;21:116. doi: 10.1186/s12891-020-3122-y (PMC7035667; doi:10.1186/s12891-020-3122-y)
Supplement: Supplementary file 1 — Additional file 1. Fluid attenuated inversion recovery fat suppressed imaging at 7T. The development of the FLAIR-FS sequence was focused at nulling the signal from intraarticular fluid in order to achieve an optimized image contrast between fluid and hyperintense synovium. In a preliminary series with a patient who was not part of the final study sample, a sequential experiment with inversion time values (TI) from 1800 to 2600 ms was performed and the most appropriate TI-value for fluid attenuation and differentiation between fluid and synovium was determined visually at 2000 ms. [file 12891_2020_3122_MOESM1_ESM.docx]

**Additional file 1**


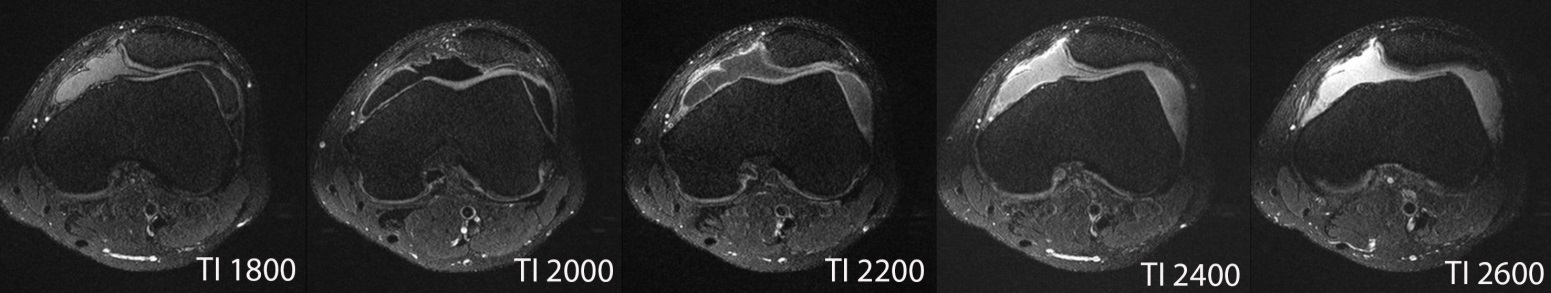


Fluid attenuated inversion recovery fat suppressed imaging at 7T. The development of the FLAIR-FS sequence was focused at nulling the signal from intraarticular fluid in order to achieve an optimized image contrast between fluid and hyperintense synovium. In a preliminary series with a patient who was not part of the final study sample, a sequential experiment with inversion time values (TI) from 1800 to 2600 ms was performed and the most appropriate TI-value for fluid attenuation and differentiation between fluid and synovium was determined visually at 2000 ms.
